# Training for Micrographia Alters Neural Connectivity in Parkinson's Disease

**DOI:** 10.3389/fnins.2018.00003

**Published:** 2018-01-19

**Authors:** Evelien Nackaerts, Jochen Michely, Elke Heremans, Stephan P. Swinnen, Bouwien C. M. Smits-Engelsman, Wim Vandenberghe, Christian Grefkes, Alice Nieuwboer

**Affiliations:** ^1^Department of Rehabilitation Sciences, KU Leuven, Leuven, Belgium; ^2^Department of Neurology, Cologne University Hospital, Cologne, Germany; ^3^Department of Movement Sciences, KU Leuven, Leuven, Belgium; ^4^Department of Health and Rehabilitation Sciences, University of Cape Town, Cape Town, South Africa; ^5^Department of Neurology, University Hospitals Leuven, Leuven, Belgium; ^6^Institute of Neuroscience and Medicine - Cognitive Neurology (INM-3), Research Centre Jülich, Jülich, Germany

**Keywords:** Parkinson's disease, micrographia, visual cueing, motor learning, dynamic causal modeling

## Abstract

Despite recent advances in clarifying the neural networks underlying rehabilitation in Parkinson's disease (PD), the impact of prolonged motor learning interventions on brain connectivity in people with PD is currently unknown. Therefore, the objective of this study was to compare cortical network changes after 6 weeks of visually cued handwriting training (= experimental) with a placebo intervention to address micrographia, a common problem in PD. Twenty seven early Parkinson's patients on dopaminergic medication performed a pre-writing task in both the presence and absence of visual cues during behavioral tests and during fMRI. Subsequently, patients were randomized to the experimental (*N* = 13) or placebo intervention (*N* = 14) both lasting 6 weeks, after which they underwent the same testing procedure. We used dynamic causal modeling to compare the neural network dynamics in both groups before and after training. Most importantly, intensive writing training propagated connectivity via the left hemispheric visuomotor stream to an increased coupling with the supplementary motor area, not witnessed in the placebo group. Training enhanced communication in the left visuomotor integration system in line with the learned visually steered training. Notably, this pattern was apparent irrespective of the presence of cues, suggesting transfer from cued to uncued handwriting. We conclude that in early PD intensive motor skill learning, which led to clinical improvement, alters cortical network functioning. We showed for the first time in a placebo-controlled design that it remains possible to enhance the drive to the supplementary motor area through motor learning.

## Introduction

Micrographia is a common disabling symptom of Parkinson's disease (PD) and is defined as “*an impairment of a fine motor skill manifesting mainly as a progressive or stable reduction in amplitude during a writing task”* (Wagle Shukla et al., 2012). Recent research highlighted the role of the posterior putamen in micrographia, as a strong correlation was found between reduced activity in this region and smaller writing sizes (Wu et al., 2016). Dopaminergic medication and deep brain stimulation only partially alleviate writing amplitude (Lange et al., 2006; Tucha et al., 2006; Bidet-Ildei et al., 2011). Therefore, non-pharmacological therapeutic supplements are needed to address this clinically relevant motor deficit. It was shown that motor learning can improve motor performance in PD, albeit with slower learning curves than in healthy controls (Nieuwboer et al., 2009; Abbruzzese et al., 2016). In addition, discrete external stimuli, or cues, have been shown to improve motor capacity in patients suffering from PD (Nieuwboer et al., 2007; Nackaerts et al., 2013; Spaulding et al., 2013). We recently demonstrated that 6 weeks of intensive visually-cued amplitude training led to robust improvements of writing (Nackaerts et al., 2016a). This randomized placebo-controlled study rendered consolidated gains, as automatization, transfer to untrained writing and 6 week-retention were demonstrated.

Still, rehabilitation is a relatively “new player” in the field of therapeutic options and the neural foundation of motor training is presently unknown in the context of neurodegenerative disease. So far, studies reporting training-dependent neural network changes addressed short-term learning (Wu et al., 2008, 2010, 2015a). More recently, long-term learning studies have provided evidence that exercise can trigger plasticity-related changes in PD (Duchesne et al., 2016; Hirsch et al., 2016; Maidan et al., 2017). Duchesne et al. (2016) demonstrated changes in functional brain activity during a serial reaction time paradigm following 12 weeks of aerobic exercise training. In addition, 6 weeks of virtual reality training targeting motor and cognitive aspects of gait and balance resulted in a decreased reliance on the frontal regions during an imagined walking fMRI task (Maidan et al., 2017). Moreover, recent research also revealed brain plasticity at the structural level in PD, which may be associated with training-induced balance improvements (Sehm et al., 2014). So far, studies failed to provide rigorous evidence for training specificity, as placebo-controlled studies were lacking (Thomas and Baker, 2013). Also, learning-related effective connectivity changes are still illusive, especially for motor skills relevant during daily life.

As such, the current study focused on cortical network shifts underlying long-term training of handwriting in a placebo-controlled design. In line with other learning studies, we chose the novel Dynamic Causal Modeling (DCM) approach to assess effective connectivity (Stephan et al., 2010; Tzvi et al., 2014, 2015; Alves-Pinto et al., 2015). We were particularly interested in connectivity with the supplementary motor area (SMA) due to its wide involvement in motor control (Nachev et al., 2008) and the known hypo-activity and connectivity in PD (Samuel et al., 1997; Sabatini et al., 2000; Herz et al., 2014a). We hypothesized that in patients receiving writing training we would find increased connectivity in the cortico-cerebellar motor networks (implicating the SMA), unlike those undergoing placebo (Wu et al., 2008, 2015b; Herz et al., 2014b; Michely et al., 2015). Furthermore, we expected similar changes in connectivity for cued and uncued handwriting after intensive writing training, as at the behavioral level improvements in amplitude were also found irrespective of cueing (Nackaerts et al., 2016a).

## Methods

### Subjects

Forty-two PD patients were included as part of a large randomized placebo-controlled study on the impact of writing training. The behavioral results of this trial were described in a previous paper (Nackaerts et al., 2016a). Earlier work by our group also compared writing-related changes at the neural network level in a partially overlapping patient group with healthy controls at the neural network level (Nackaerts et al., 2018). All participants were right-handed, as determined by the Edinburgh handedness scale (Oldfield, 1971). Inclusion criteria consisted of: (i) diagnosis according to the Brain Bank criteria (Hughes et al., 1992); (ii) Hoehn & Yahr (H&Y) stage I to III while on medication (Hoehn and Yahr, 1967); (iii) right disease-dominance in patients in H&Y I; and (iv) presence of micrographia defined by a score > 1 on item II.7 of the MDS Unified Parkinson's Disease Rating Scale (MDS-UPDRS) (Goetz et al., 2008). Exclusion criteria were: (i) Mini-Mental State Examination (MMSE) < 24 (Folstein et al., 1975); (ii) visual impairments that could not be corrected by glasses; (iii) other upper limb problems impeding handwriting; and (iv) contra-indications for MRI. After screening, 9 patients of the larger cohort did not meet inclusion criteria for the MRI study. Five additional patients were excluded due to excessive head movement and one because time-series for effective connectivity analysis could not be extracted (see below). This resulted in the inclusion of 27 patients in the final task-related functional imaging analysis on the effects of training (*N*_experimental_ = 13, *N*_placebo_ = 14), reported here for the first time.

The local Ethics Committee of the University Hospitals Leuven approved the study in accordance with the Declaration of Helsinki. Written informed consent was obtained prior to participation and after explanation of the protocol. The trial was registered as ClinicalTrials.gov Protocol Record G.0906.11.

### Study design

Using a stratified randomization procedure based on H&Y stage (I–III) and age (≤ 65 or > 65 years), patients were assigned to a writing training supported by external visual cueing (= experimental) or a stretch and relaxation program (= placebo). Writing training consisted of progressive exercises to maintain writing amplitude with the help of visual cues (Nackaerts et al., 2016a). The placebo program aimed to teach patients how to relax in general and alleviate tension in the upper limbs (Nackaerts et al., 2016a). Exercises were performed while lying down or sitting and consisted of breathing exercises, progressive relaxation, mindfulness, and yoga. Both training groups received an equally time-intensive therapy, with the same number of sessions (5x/week for 6 weeks) and the same duration of each session (30 min). Both groups also had an equal amount of contact with and supervision by the therapist. Similar expectations were created by suggesting that the aim of treatment was to improve motor performance through taking away tension.

Patients were tested twice, at baseline and after 6 weeks of training, and this both outside and inside the scanner (Supplementary Figure 1). Additionally, a practice session in a dummy scanner was organized for all participants to become acquainted with the protocol. Medication intake was kept constant throughout the study and testing occurred while on dopaminergic medication. Patients were tested ~1 h after the last medication intake and time of testing was standardized for both time points.

### Behavioral assessment

For the present study, the primary writing outcome in- and outside the scanner comprised a simple repetitive pre-writing task of making three loops, similar to the letter “e,” with the right hand on a touch-sensitive writing tablet from the bottom of the blue to the top of the yellow target zone (Supplementary Figure 2) (Nackaerts et al., 2016a). After completion of the third loop, participants had to return to the start circle via the gray zone. Each loop-sequence disappeared from the screen when re-entering the start circle, allowing continuous repetition of the same figure without hand repositioning movements until the end of the 27 s trial (Nackaerts et al., 2016b). The distance between the bottom of the blue and top of the yellow target zone was 0.6 cm. The cued writing task was performed in the presence of these colored target zones, while in the uncued condition the target zones disappeared after 1.5 s. To assess daily life handwriting, we used the “Systematic Screening of Handwriting Difficulties (SOS)” test, involving writing a text on paper for 5 min continuously (Nackaerts et al., 2017).

In the scanner, the same pre-writing test was assessed using a custom-made MRI-compatible tablet (Supplementary Figure 1). Participants performed the three-loop-sequence described above with real-time visual feedback of what was written provided via a double mirror built into the head coil. A pacing tone was used to standardize performance, i.e., participants were expected to complete one loop sequence in 2 s. Each of the two conditions (cued—uncued) lasted 27 s, was preceded by a rest period of 6 s and an instruction of 3 s, and repeated four times within one run in random order. All participants performed three runs. Of five patients (one experimental and four placebo) only two runs could be included due to excessive head movements.

Additionally, all patients underwent a clinical test battery, including the MDS-UPDRS-III and H&Y staging scale (Hoehn and Yahr, 1967; Goetz et al., 2008) and calculation of the Levodopa Equivalent Dose (LED) (Tomlinson et al., 2010). Fine motor skills were assessed by means of the Manual Ability Measure (MAM-16) questionnaire (Chen et al., 2005). Cognitive abilities were evaluated using the MMSE (Folstein et al., 1975) and emotional status using the Hospital Anxiety and Depression Scale (HADS) (Zigmond and Snaith, 1983).

### Functional MRI acquisition and preprocessing

Imaging was carried out in a Philips Achieva 3T scanner (Best, The Netherlands). A standard head coil was used with foam padding to restrict head motion. High-resolution T1-weighted anatomical scans [T1 Turbo Field Echo (TFE) sequence, duration = 383 ms; slice number = 182; slice thickness = 1.2 mm; time repetition (TR) = 9.624 s; time echo (TE) = 4.6 ms; flip angle = 8°; matrix = 256 × 256; FOV = 218.4 × 250 × 250 mm] and T2-weighted functional images were acquired for each participant using gradient echo-planar imaging (EPI) pulse sequence (50 transversal slices, slice thickness = 2.5 mm, slice gap = 0.25 mm, TE = 30 ms, TR = 3000 ms, flip angle = 90°, matrix = 80 × 80).

Functional imaging data were pre-processed using SPM8 (Wellcome Department of Imaging Neuroscience, University College London, UK) implemented in Matlab (R2011a). All functional images were realigned to the reference (mean) image and co-registered to each subject's T1 anatomical image. All images were normalized to Montreal Neurological Institute (MNI) space using the segmented anatomical image and smoothed with a 6-mm full width at half maximum Gaussian kernel. Differences in head motion parameters between groups were tested using the framewise displacement method (Power et al., 2012). There was no difference between the experimental and placebo group at baseline (*p* = 0.940) and post-training (*p* = 0.952) or between baseline and post-training in the experimental (*p* = 0.920) and placebo group (*p* = 0.900).

### Brain activity analysis

Data were analyzed using the general linear model approach in SPM8. Both experimental conditions (cued—uncued) were modeled and head motion parameters were added as covariates of no interest to correct for confounding effects. Basic main effects for both conditions were calculated for each participant. These individual contrasts were entered in a second-level ANOVA using a full factorial design with the factors TRAINING (experimental—placebo), TIME (pre—post) and CONDITION (cued—uncued), with MAM-16 as a covariate. *Post-hoc* t-tests were performed to explore the main differences and interactions (*p* < 0.05, FWE-corrected).

### Dynamic causal modeling

As our main interest concerned the directed influence of one brain region over another, i.e., effective connectivity, we used DCM as our main analysis method (Friston et al., 2003). DCM is a Bayesian inference method that relies on *a priori* defined hypothesis-driven neuronal models of interacting brain regions, relevant to a specified task or pathology. DCM does not explore all possible models, but starts with defining the relevant regions of interest (ROIs) and the connections, pertaining to specific hypotheses.

#### Region of interest selection

**Figure 1:**
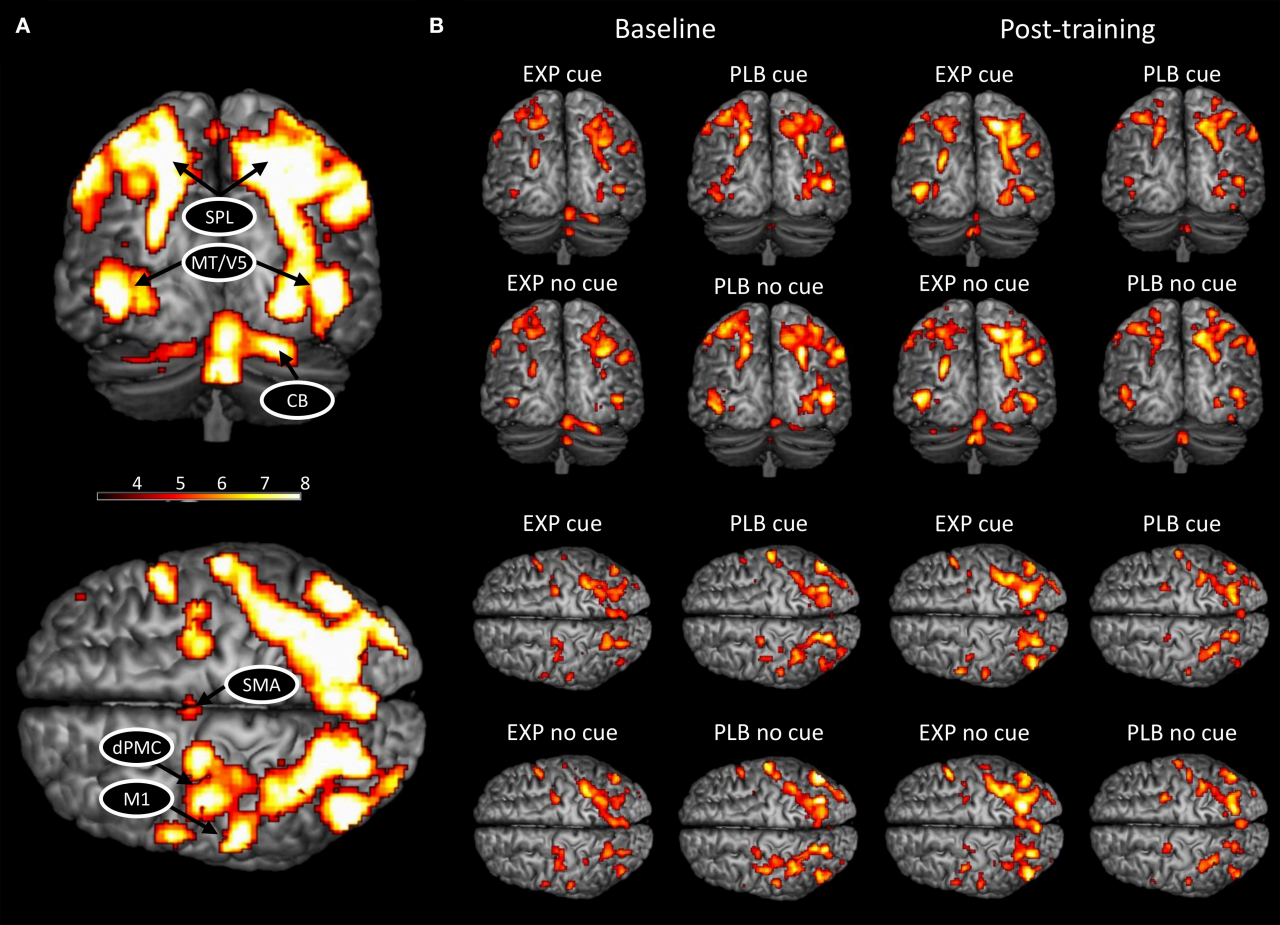
BOLD activation pattern during handwriting at baseline and post-training. **(A)** Activated network for both conditions combined; **(B)** Activated network in each condition and group separately at baseline and post-training. CB, cerebellum; HC, healthy controls; PD, Parkinson's disease; dPMC, dorsal premotor cortex; M1, primary motor cortex; SMA, supplementary motor area; SPL, superior parietal lobe; MT/V5, motion sensitive middle temporal visual area. The threshold was set at *p* < 0.001 (uncorrected) to achieve better visualization of all areas.

In the present study, the ROIs were selected based on their known involvement in handwriting (Horovitz et al., 2013; Planton et al., 2013), motor learning (Doyon et al., 2009; Dayan and Cohen, 2011; Hardwick et al., 2013), and altered activation and connectivity patterns in PD (Samuel et al., 1997; Sabatini et al., 2000; Herz et al., 2014a; Wu et al., 2015b). We also only included areas that were activated in each condition (Figure 1). This resulted in the inclusion of bilateral motion sensitive Middle Temporal visual area (MT/V5), bilateral superior parietal lobe (SPL), left primary motor cortex (M1), left dorsal premotor cortex (dPMC), left SMA, and right cerebellar lobule VI.

Next, we extracted the first eigenvariate of the blood-oxygen-level dependent (BOLD) time-series adjusted for effects of interest from the eight ROIs at subject-specific coordinates. ROIs were defined as spheres (4 mm radius) centered upon individual activation maxima based on individually normalized SPMs (threshold *p* < 0.001; in case of non-significant voxels, the threshold was lowered to *p* < 0.05) (Supplementary Table 1). For post-training analyses, the same ROIs were included and a deviation of maximally 4 mm from baseline coordinates was allowed to guarantee spatial consistency of anatomical areas.

#### Connectivity models

**Figure 2:**
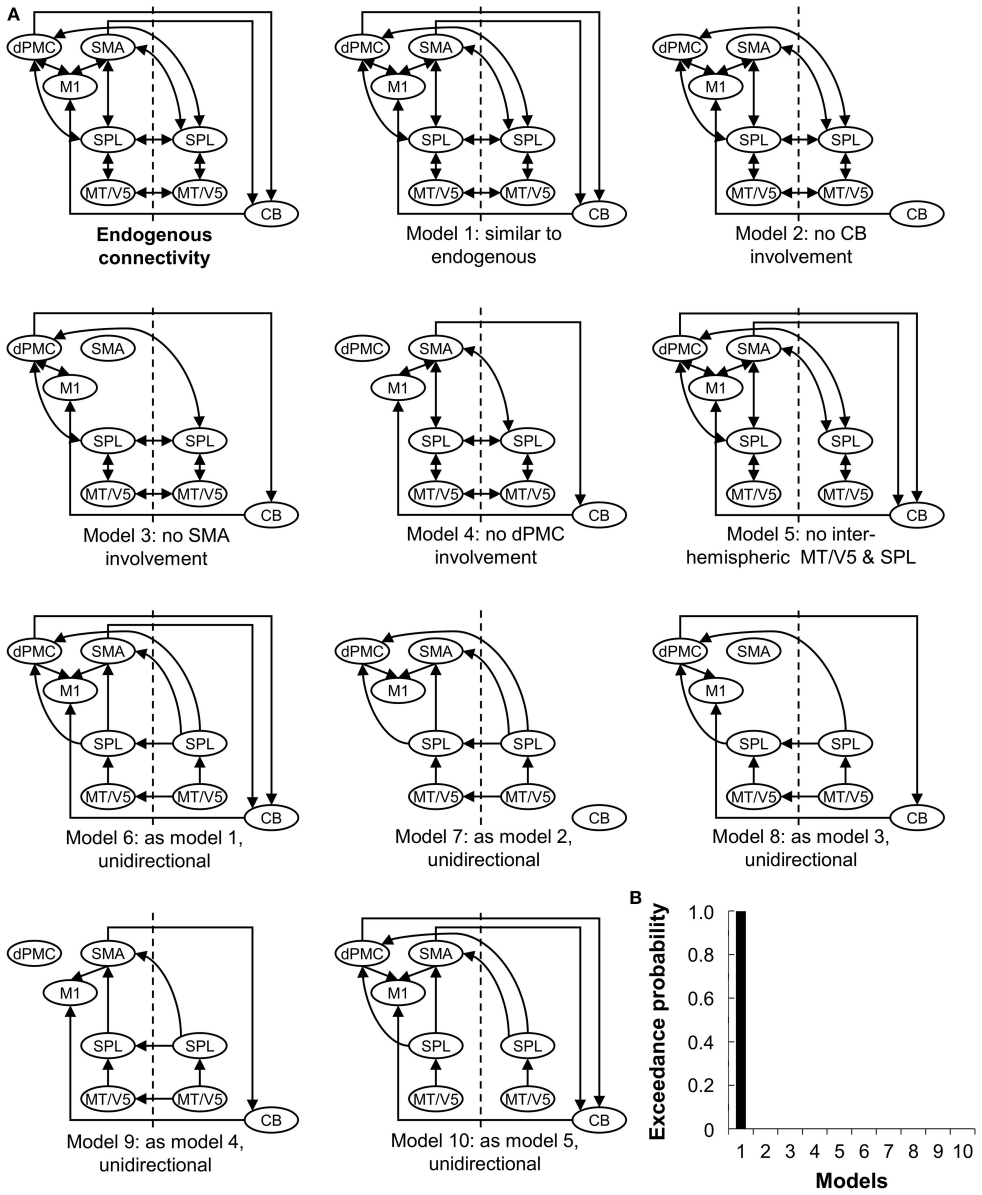
Ten models compared using Bayesian Model Selection. **(A)** Model 1-10 represent modulations of the connections (DCM-B). The input was set at bilateral V5. CB, cerebellum; CT, healthy controls; PD, Parkinson's disease; dPMC, dorsal premotor cortex; M1, primary motor cortex; SMA, supplementary motor area; SPL, superior parietal lobe; MT/V5, motion sensitive middle temporal visual area. **(B)** Bayesian model selection comparing the 10 models for patients at baseline and post-training.

The endogenous structure of the network (DCM-A) was based on previous studies on effective connectivity of the extended motor system (Grefkes et al., 2010; Michely et al., 2015; Wu et al., 2015b). Given that all patients wrote with their right hand, motor areas in the left hemisphere were assumed to be involved (Herz et al., 2014a), resulting in the inclusion of left M1, dPMC, and SMA. In addition, the right cerebellum was included in line with the literature (Michely et al., 2015; Wu et al., 2015b). We included the connections between these areas based on studies by Michely et al. (2015) and Wu et al. (2015b). Furthermore, bilateral MT/V5 and SPL were assumed to play an important role, as the dorsal visual stream is crucial for visuomotor integration (Kravitz et al., 2011), such as needed for handwriting. Connections between MT/V5 and SPL and from SPL to the motor areas were assumed based on the work of Grefkes et al. (2010). Given the role of the right hemisphere in processing visual stimuli (Woolley et al., 2010), we finally hypothesized that the interhemispheric connections between MT/V5 and SPL were meaningful to include in the model. Secondly, we set up alternative models of varying complexity representing biologically plausible hypotheses on how connectivity might be modulated depending on the experimental conditions, i.e., visual cueing and learning (DCM-B) (Figure 2A), which led to the construction of 10 models with the same endogenous structure. Concerning automatization and learning of a motor skill, Wu et al. highlighted the compensatory roles of dPMC and cerebellum, to counterbalance the reduced activation and connectivity in the basal ganglia and SMA in PD (Wu and Hallett, 2005; Wu et al., 2010, 2011, 2015b). In addition, several studies suggested a specialized role for dPMC and SMA in the external and internal control of movement (Jueptner and Weiller, 1998; Jenkins et al., 2000; Debaere et al., 2003), while others hypothesized that this specialization might not be straightforward (te Woerd et al., 2015). Therefore, models 1-4 were included to assess the differing roles of dPMC, SMA and cerebellum in motor learning and cueing by systematically excluding these areas from the models. Model 5 assessed the necessity of interhemispheric connections for visual cueing, as processing of visual stimuli was specifically attributed to the right hemisphere (Woolley et al., 2010) and external control of movement to the additional activation in visual areas (Gowen and Miall, 2007). The complexity of the models was finally addressed in models 6-10 by limiting the connectivity to forward connections. Due to the importance of MT/V5 in the processing of visual information to guide movement (Debaere et al., 2003), we set the driving input (DCM-C) on this area across conditions for all models.

Subsequently, Bayesian model selection (BMS) was used to identify the model with the highest probability, using a random effects approach (Stephan et al., 2009). This statistical method determines the probability of a certain dataset depending on the proposed models. A good model will explain the data as well as possible, while guaranteeing minimal complexity (Stephan et al., 2010). The most likely model was identified by taking into account the exceedance probability for the model-set, capturing the greatest likelihood to have generated the observed BOLD signal. BMS was followed by inference on model parameters, by extraction of the coupling estimates of the winning model for each participant.

### Statistical analysis

#### Statistical analysis of general characteristics

Depending on the data distribution and equality of variances, either a parametric independent samples t-test or non-parametric Mann-Withney *U*-test was performed to compare both patient groups. For gender, most affected side and H&Y stage, a Chi-squared test was performed.

#### Processing and statistical analysis of handwriting performance

Data from the touch-sensitive tablet were filtered at 7 Hz with a 4th-order Butterworth filter (Van Gemmert et al., 2003) and processed using Matlab (R2011b; The Mathworks Ltd., US). The primary outcome variable was change in writing amplitude, expressed as a percentage of change relative to baseline performance. Additionally, change in variability of amplitude (COV_Ampl_) and writing speed were also assessed. Statistical analysis was performed using SPSS (version 24, IBM, US), for writing performance in and outside the scanner separately. A mixed model ANOVA was computed with TRAINING (experimental—placebo) as a between-subject factor and CONDITION (cued—uncued) as a within-subject factor, with MAM-16 as a covariate in line with the behavioral study (Nackaerts et al., 2016a). A Greenhouse-Geisser correction was applied to all analyses as the assumption of sphericity was violated. Partial eta squared (η^2^) was calculated as a measure of effect size. The significance level was set at *p* < 0.05.

A blinded researcher evaluated the SOS-test manually. Mean writing size (mm) and writing velocity (letters written in 5 min) were determined. The total SOS-score, representing quality, was determined and consisted of: (i) fluency of letter formation; (ii) fluency in connections between letters; (iii) regularity of letter height; (iv) space between words; and (v) straightness of the sentences (Nackaerts et al., 2017). A higher total SOS-score indicated worse quality of handwriting (0–10). The change in size, velocity and quality were compared using an independent samples *t*-test. Cohen's d was calculated as a measure of effect size.

#### Statistical analysis of connectivity data

A mixed model ANOVA was performed on the coupling estimates with TRAINING (experimental - placebo) as a between-subject factor and CONDITION (cued—uncued), TIME (pre—post), and CONNECTION as within-subject factors and with MAM-16 as a covariate. Only connections that survived a Bonferroni-corrected 1-sample *t*-test for the entire group of participants were included (taking into account the number of connections) (see Supplementary Table 2 for connections included in each analysis) and Greenhouse-Geisser corrections were applied. Finally, a partial correlation analysis was performed between coupling estimates of altered connections and changes in performance due to training. The significance level was set at *p* < 0.05.

Even though there were no statistically significant differences between both patient groups for MDS-UPDRS-III (experimental: 27.3 ± 14.1; placebo: 21.8 ± 8.4) and LED (experimental: 498.8 ± 313.8 mg/24 h; placebo: 449.5 ± 337.3 mg/24 h), the same analyses (handwriting performance and connectivity data) were performed adding both as additional covariates to explore potential effect of disease severity and/or medication intake.

## Results

### Analysis with MAM-16 as covariate

#### Behavioral data

Table 1 displays the well-matched clinical characteristics of the placebo and training group. During scanning, no significant differences in writing behavior were found. Outside the scanner, a significant main effect of GROUP revealed a greater change in amplitude relative to baseline performance in the experimental (13.7%, value corrected for MAM-16) compared to placebo group (−0.6%, value corrected for MAM-16), irrespective of cues (*F* = 4.420; *p* = 0.046; η^2^ = 0.156). COV_Ampl_ and speed did not differ. A similar result was found for writing amplitude on paper, with a greater change in SOS size in the experimental (15.3%) compared to placebo group (−5.8%) (*t* = 2.621; *p* = 0.015; *d* = 1.006). No significant differences were found for SOS score and velocity.

**Table 1:** General characteristics.

	**EXP (*N* = 13)**	**PLB (*N* = 14)**	***p***
Age (years)	63.1 ± 7.8	62.1 ± 8.3	0.766
Gender* (♂/♀)	7/6	10/4	0.345
EHI (%)	100.0 (90.0, 100.0)	95.0 (89.9, 100.0)	0.685
MMSE (0–30)	29.0 (29.0, 30.0)	29.0 (28.0, 30.0)	0.402
MAM-16 (0–64)	55.1 ± 5.0	58.1 ± 4.3	0.104
HADS-Anxiety (0–21)	6.4 ± 4.1	4.9 ± 4.1	0.366
HADS-Depression (0–21)	4.9 ± 3.8	3.6 ± 2.6	0.519
Disease duration (years)	6.0 ± 4.1	4.1 ± 2.8	0.180
MDS-UPDRS-III (0–132)	27.2 ± 14.1	21.8 ± 8.4	0.231
MDS-UPDRS-III UL (0–56)	13.6 ± 6.9	11.6 ± 5.5	0.401
Most affected side UL (R/L)*	9/4	7/7	0.310
H&Y (1–5)*	2.0 (2.0, 2.0)	2.0 (2.0, 2.0)	0.793
LED (mg/24 h)	600.0 (177.5, 697.5)	347.5 (195.0, 652.5)	0.685

Mean ± standard deviation is presented in case of normal distribution and equality of variances (independent samples t-test), otherwise Median (first, third quartile) is displayed (Mann-Withney U-test). ^*^Indicates variables analyzed using the Chi-squared test. EHI, Edinburgh Handedness Inventory; EXP, experimental writing training; HADS, Hospital Anxiety and Depression Scale; H&Y, Hoehn & Yahr stage; LED, Levodopa Equivalent Dose; MAM-16, Manual Ability Measure; MDS-UPDRS-III, MDS Unified Parkinson's Disease Rating Scale part III; MMSE, Mini Mental State Examination; PLB, placebo training; UL, upper limb items.

#### Neural activation pattern

Similar networks were activated post-training compared to baseline (Figure 1). A significant main effect of cue was found (FWE-corrected, *p* < 0.05), with an increased BOLD activity during cued writing in bilateral visual cortex and an increased activation of right cerebellum lobule VI in the uncued condition (Table 2). No main effects for group and time or interactions were found.

**Table 2:** Difference in BOLD activation between cued and uncued handwriting.

**Brain region**	**Coordinates**			***z*-value**	**K_E_**
	***X***	***Y***	***Z***		
**With** > **without cue**					
Right calcarine	15	−90	0	Inf	236
Left mid occipital	−15	−88	−6	Inf	199
Left mid occipital	−33	−88	14	6.119	56
Left fusiform	−29	−76	−10	5.328	23
**Without** > **with cue**					
Right cerebellar lobule VI	13	−58	−20	5.917	54

*P < 0.05 FWE-corrected, voxel threshold = 20*.

#### Bayesian model selection

Random-effects BMS revealed Model 1 as the most likely model explaining the data for the experimental and placebo group (both exceedance probability > 87%) (Figure 2B).

#### Connectivity analysis

**Figure 3:**
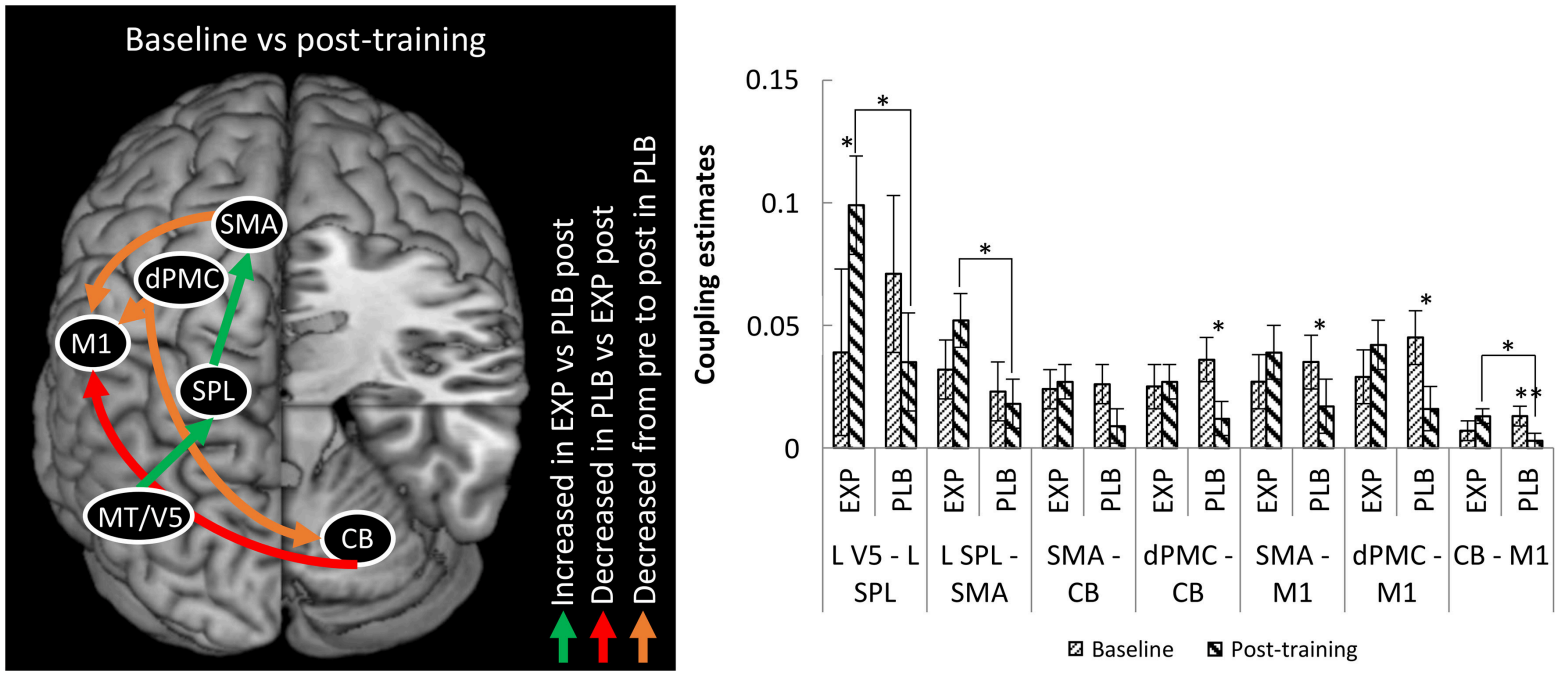
Differences in connectivity between EXP and PLB and from baseline to post-training. Only excitatory connections are displayed, all corrected for MAM-16. CB, cerebellum; EXP, experimental group; PLB, placebo group; dPMC, dorsal premotor cortex; M1, primary motor cortex; SMA, supplementary motor area; SPL, superior parietal lobe; MT/V5, motion sensitive middle temporal visual area. (^*^p < 0.05; ^**^p < 0.01) Error bars represent standard errors.

Regarding endogenous connectivity (DCM-A), no significant main effects or interactions were found. The mixed ANOVA for condition-specific connectivity (DCM-B) revealed a significant TRAINING x TIME x CONNECTION interaction (*F* = 2.473; *p* = 0.035; η^2^ = 0.093). *Post-hoc* analysis revealed a stronger positive influence of left MT/V5 on SPL (*p* = 0.039) and of left SPL on left SMA (*p* = 0.034) in the experimental group compared to placebo post-training (Figure 3). This was accompanied by a significant increase from baseline to post-training in the experimental group for the former connection (*p* = 0.042). In summary, writing training enhanced connectivity via the left hemispheric visuomotor stream to the SMA relative to placebo.

For the connection from right cerebellum to left M1 there was stronger connectivity in the experimental compared to the placebo group post-training (*p* = 0.030), though this result was driven by a decrease in connection strength from baseline to post-training in the placebo group (*p* = 0.006). In the connections from left dPMC to right cerebellum and from left dPMC and SMA to left M1 there was also decreased coupling in this group (resp. *p* = 0.029; *p* = 0.014 and *p* = 0.047). No significant correlations between behavioral and connectivity parameters were found.

### Analysis with MDS-UPDRS-III and LED as additional covariates

#### Behavioral data

During scanning, no significant differences in writing behavior were found. Outside the scanner, a tendency toward a main effect of GROUP revealed a greater change in amplitude relative to baseline performance in the experimental (12.7%) compared to placebo group (0.4%), irrespective of cues (*F* = 3.596; *p* = 0.071). COV_Ampl_ and speed did not differ.

#### Connectivity analysis

Regarding endogenous connectivity (DCM-A), no significant main effects or interactions were found. The mixed ANOVA for condition-specific connectivity (DCM-B) revealed a significant TRAINING x TIME x CONNECTION interaction (*F* = 2.324; *p* = 0.049). *Post-hoc* analysis revealed a stronger positive influence of left MT/V5 on SPL (*p* = 0.031) and of left SPL on left SMA (*p* = 0.011) in the experimental group compared to placebo post-training. This was accompanied by an increase from baseline to post-training in the experimental group for the former connection (*p* = 0.059). In summary, writing training enhanced connectivity via the left hemispheric visuomotor stream to the SMA relative to placebo.

For the connection from right cerebellum to left M1 there was stronger connectivity in the experimental compared to the placebo group post-training (*p* = 0.019), though this result was driven by a decrease in connection strength from baseline to post-training in the placebo group (*p* = 0.006). In the connections from left dPMC and SMA to right cerebellum and from left dPMC and SMA to left M1 there was also decreased coupling in this group (resp. *p* = 0.022; *p* = 0.039; *p* = 0.035, and *p* = 0.051).

## Discussion

In this study, we showed for the first time that writing training modulated neural connectivity between task-related cortical regions in PD patients. In our earlier work, we found reduced visuo-parietal connectivity in the right hemisphere in PD patients compared to healthy elderly (Nackaerts et al., 2018). After intensive handwriting training with cues, micrographia was alleviated and a stronger coupling in the visuomotor stream of the left hemisphere emerged. These results were placebo-controlled and cue-independent, testifying the strength of these findings.

Patients with PD who received prolonged and intensive writing training displayed significant connectivity changes within the handwriting network, as the connectivity targeting the SMA increased through the influence of an enhanced left-hemispheric visuo-parietal coupling. The SMA plays an important role in the preparation and execution of movements, specifically of internally controlled movements and those involving bilateral coordination (Goldberg, 1985; Jueptner et al., 1997; Jenkins et al., 2000; Debaere et al., 2003). A more recent view propagates the role of the SMA as the interface of cognitive-action association (Nachev et al., 2008). As the SMA is a major output region of the basal ganglia, poor functioning of the SMA and basal ganglia was shown to result in difficulties with the execution of voluntary, automatic movements (Wu et al., 2015a), such as handwriting in PD. It has been proposed that strengthening the coupling between two areas is indicative of communication that is more efficient and that this proficient neural processing is characteristic for motor learning (Wu et al., 2010; Alves-Pinto et al., 2015). As such, the current result indicates that cued amplitude training resulted in more strongly visually controlled movement execution. The increased visual steering probably appealed to connections in the left visuomotor integration system, since baseline comparison between patients and controls implied that these connections were unaffected by the disease (Nackaerts et al., 2018). The fact that this led to greater involvement of the SMA is an important finding, given the consistently reported reduced neural coupling with this structure in PD (Wu et al., 2011, 2016). Furthermore, increased coupling with the SMA was reported previously to occur after administration of dopaminergic medication (Herz et al., 2014b; Michely et al., 2015). Finally, the importance of re-involving the SMA was stressed by studies in healthy adults, which consistently reported increased SMA-participation as learning progressed (Jenkins et al., 1994; Karni et al., 1995; Toni et al., 1998; Ungerleider et al., 2002; Lehericy et al., 2005). More recent studies expanded this view, as the SMA was found to play a specific role in the stabilization of motor memories and sleep-dependent consolidation (Tanaka et al., 2010; Tamaki et al., 2013). As such, our results support a similar trend in the relearning of motor skills in PD. By means of consolidating a goal-directed mode of motor control (writing with visual targets), re-involvement of the SMA was achieved via alternative pathways. Moreover, these results suggest that focused rehabilitation can alter connectivity patterns similar to that of pharmacological treatment, leading to clinical improvements demonstrated outside the scanner.

Contrary to our hypothesis, connection strength in the SMA-M1-cerebellum loop did not increase after training. This hypothesis was based on an earlier study of learning a visuomotor association task when off medication (Wu et al., 2015b). The different nature of the present task and the fact that scanning occurred while on medication, possibly explain this conflicting result. Despite these dissimilarities, Wu et al. found that a continued drive from the DLPFC onto SMA and PMC was necessary to achieve a degree of automaticity in PD (Wu et al., 2015b), stressing the importance of recruiting the SMA via alternative, possibly task-dependent pathways.

No evidence of cue-dependency was found at the behavioral level, nor at the effective connectivity level. Patients improved writing amplitude outside the scanner and presented with stronger effective connectivity regardless of the presence of cues after training. This is also in line with our behavioral results, which showed robust transfer of learned skills to conditions different from the learning context (Nackaerts et al., 2016a). In young healthy adults, however, training of a bimanual coordination task with visual feedback revealed sustained activation of visual areas and a deterioration of performance when feedback was withdrawn (Ronsse et al., 2011). The fact that this was not found in the current study is possibly the result of the gradual reduction in the size of the target zones during training.

A final finding was a decrease in connection strength among dPMC, SMA, cerebellum, and M1 in the placebo group, which coincided with a decrease in writing amplitude. One possible explanation is that the increased arousal caused by the novelty of the scanner at baseline led to a better performance during the first scan compared with the second scan, also known as the Hawthorne effect (McCarney et al., 2007). Patients with PD might be specifically prone to this effect (Robles-Garcia et al., 2015). Wu et al. (2015b) showed that attention had a significant effect on the connectivity of the PMC, cerebellum, and DLPCF in PD. As such, the combination of reduced arousal/attention and the lack of training effects in the placebo group could explain the decrease in connection strength in the above-mentioned motor network, resulting in poorer motor performance. We did not see this reduction in connection strength in the group receiving writing training, possibly due to the maintained focus as a result of the visually-cued training.

### Interpretational issues

Patients were deliberately tested while on dopaminergic medication to match training conditions with real-life rehabilitation lasting for 6 weeks. Importantly, previous research exposed significant effects of dopaminergic medication on SMA activity and connectivity (Haslinger et al., 2001; Herz et al., 2014b; Michely et al., 2015) and of disease progression on SMA connectivity in patients with PD (Wu et al., 2011). However, these findings pertained to altered motor performance, rather than motor learning. So far, studies showed that dopaminergic medication can have a negative effect on motor learning by means of a dopamine overdose in the ventral putamen (Kwak et al., 2010, 2012; Vaillancourt et al., 2013) and that motor learning capacity could be associated with disease progression (Stephan et al., 2011; Dan et al., 2015). Given clinically relevant differences in LED dose and MDS-UPDRS-III score, we deemed it necessary to exclude possible effect of dopaminergic medication or disease severity on our results, even though there were no significant differences. Adding both as additional covariates did not alter our results. However, further research is necessary to look into the influence of dopaminergic medication and disease severity on effective connectivity within the cortico-striatal network in relation to motor learning.

We also ensured that no behavioral differences presented during scanning between patient groups. Price and Friston (2002) emphasized that such similar performance is necessary to enable a significant comparison at the neural level. Finally, due to the exclusion of several patients, the remaining sample size was relatively small, which compromised the statistical power of the study. Hence, the lack of brain-behavior correlations, which hampers a firm interpretation of our findings, could be resolved by including a larger sample size in future studies. As suggested by Tzvi et al. (2014) future research should also incorporate behavioral parameters into the models to address this issue. Contrary to the only other MRI study focusing on a prolonged and specific rehabilitation intervention in PD (Maidan et al., 2017), we did not find strong group differences when looking at the BOLD-activity *per sé*. One possible explanation for this is the difference between the patient groups included, which in the latter study involved more subjects with more severe PD, a longer disease duration and higher LED dose (Maidan et al., 2017).

## Conclusions

Overall, the present study adds to existing knowledge on the neural imprint of long-term motor training in PD, as we found that in contrast to placebo, successful training of writing amplitude was associated with more efficient coupling in the left visuomotor network. This cortical reorganization may have supported the robust evidence of clinical benefits of writing found outside the scanner, providing evidence for motor learning induced brain plasticity in PD.

## Author contributions

EN, EH, SS, BS-E, WV, and AN: Substantial contributions to the conception or design of the work. EN, EH, JM, CG, and AN: The acquisition, analysis, or interpretation of data for the work. EN: Drafting the work. JM, EH, SS, BS-E, WV, CG, and AN: Revising the work critically for intellectual content. EN, JM, EH, SS, BS-E, WV, CG, and AN: Final approval of the version to be published. EN, JM, EH, SS, BS-E, WV, CG, and AN: Agreement to be accountable for all aspects of the work.

### Conflict of interest statement

The authors declare that the research was conducted in the absence of any commercial or financial relationships that could be construed as a potential conflict of interest.
