# Supplementary material for: Training for Micrographia Alters Neural Connectivity in Parkinson's Disease
Source: Front Neurosci. 2018 Jan 19;12:3. doi: 10.3389/fnins.2018.00003 (PMC5780425; doi:10.3389/fnins.2018.00003)
Supplement: Supplementary file 1 [file Presentation1.PDF]

Supplementary Material

Training for micrographia alters neural connectivity in Parkinson's disease

Evelien Nackaerts*, Jochen Michely, Elke Heremans, Stephan P. Swinnen, Bouwien C. M. Smits-Engelsman, Wim Vandenberghe, Christian Grefkes, Alice Nieuwboer

*** Correspondence:** Evelien Nackaerts: evelien.nackaerts@kuleuven.be

1 Supplementary Figures and Tables

1.1 Supplementary Figures

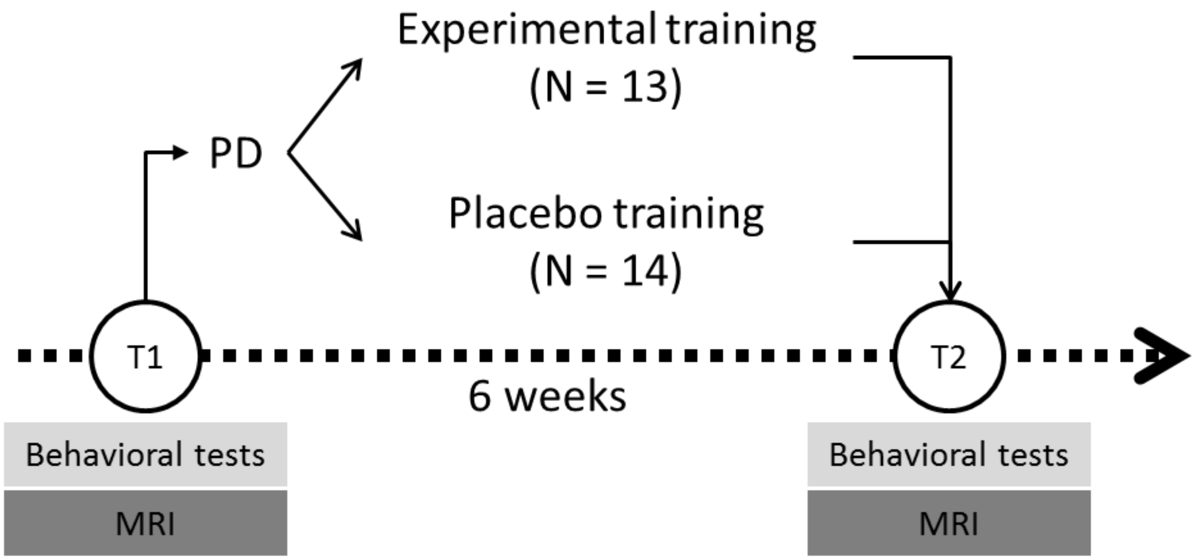

Supplementary Figure 1. Study design. T1 = baseline; T2 = post-training; Experimental training = intensive writing program; Placebo training = stretch and relaxation program.

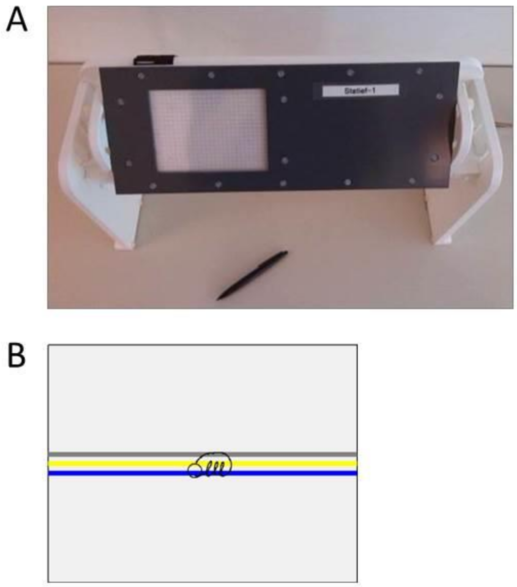

Supplementary Figure 2. MRI-compatible writing tablet (A) and writing task (B).

1.2 Supplementary Tables

Supplementary Table I: ROI coordinates for DCM analysis

	Baseline			Post-training		
	**X**	**Y**	**Z**	**X**	**Y**	**Z**
**L M1**	-35.7 ± 4.5	-25.9 ± 3.6	61.3 ± 4.2	-35.5 ±4.4	-24.9 ± 3.5	60.9 ± 4.5
**L dPMC**	-23.9 ± 2.2	-8.0 ± 3.6	55.3 ± 4.2	-23.7 ± 2.8	-7.3 ± 3.8	56.5 ± 3.9
**L SMA**	-5.8 ± 1.4	-6.0 ± 3.8	60.9 ± 5.6	-5.5 ± 1.9	-6.4 ± 4.3	61.3 ± 5.6
**L SPL**	-23.9 ± 4.2	-60.2 ± 3.4	60.6 ± 3.4	-23.7 ± 4.7	-60.0 ± 4.0	60.6 ± 3.2
**R SPL**	21.6 ± 3.9	-64.7 ± 4.3	59.4 ± 3.5	21.6 ± 3.8	-64.5 ± 4.2	59.2 ± 3.6
**L MT/V5**	-46.9 ± 3.4	-72.4 ± 4.7	1.6 ± 4.6	-46.9 ± 3.1	-72.6 ± 4.7	1.8 ± 4.6
**R MT/V5**	47.9 ± 3.5	-67.4 ± 3.7	2.3 ± 3.5	46.9 ± 3.7	-67.9 ± 3.8	1.9 ± 4.2
**R CB**	28.1 ± 3.7	-50.6 ± 4.2	-26.6 ± 3.3	28.6 ± 3.9	-51.0 ± 4.7	-26.4 ± 3.4

The group coordinates are presented as mean ± standard deviation. **Abbreviations:** CB = cerebellum; dPMC = dorsal premotor cortex; L = left; M1 = primary motor cortex; MT/V5 = motion sensitive middle temporal visual area; R = right; SMA = supplementary motor area; SPL = superior parietal lobe.

**Supplementary table II: connections that survived the Bonferroni corrected 1-sample t-test**		
**Connections**	**DCM-A**	**DCM-B**
**L MT/V5 – R MT/V5**	Included	NS
**L MT/V5 – L SPL**	Included	Included
**R MT/V5 – L MT/V5**	Included	NS
**R MT/V5 – R SPL**	Included	Included
**L SPL – L MT/V5**	NS	Included
**L SPL – R SPL**	Included	Included
**L SPL – L dPMC**	Included	Included
**L SPL – L SMA**	Included	Included
**R SPL – R MT/V5**	NS	Included
**R SPL – L SPL**	Included	Included
**R SPL – L dPMC**	Included	Included
**R SPL – L SMA**	Included	NS
**L dPMC – L SPL**	Included	Included
**L dPMC – R SPL**	Included	Included
**L dPMC – L M1**	Included	Included
**L dPMC – R CB**	Included	Included
**L SMA – L SPL**	Included	Included
**L SMA – R SPL**	Included	Included
**L SMA – L M1**	Included	Included
**L SMA – R CB**	Included	Included
**L M1 – L dPMC**	NS	NS
**L M1 – L SMA**	NS	NS
**R CB – L M1**	Included	Included

Abbreviations: CB = cerebellum; dPMC = dorsal premotor cortex; L = left; M1 = primary motor cortex; MT/V5 = motion sensitive middle temporal visual area; NS = not significant; R = right; SMA = supplementary motor area; SPL = superior parietal lobe
